# Tympanic Membrane Involvement of Multiple Myeloma: Report of a Case

**DOI:** 10.3390/hematolrep18040055

**Published:** 2026-08-04

**Authors:** Elisavet Manaki, Antri Polyviou, Sosana Delimpasi, Mathilde Chroni, Ioannis Vlastos

**Affiliations:** 1 Department of ENT, Evangelismos General Hospital, 10676 Athens, Greece; elisamanaki@gmail.com (E.M.);; 2 Department of Hematology and Lymphoma, Evangelismos General Hospital, 10676 Athens, Greece

**Keywords:** extramedullary disease, multiple myeloma, temporal bone, plasmacytoma, tympanic membrane

## Abstract

Extramedullary Involvement (EMI) in Multiple Myeloma (MM) is a rare but aggressive entity that usually occurs in advanced or relapsed cases of MM. Although otologic manifestations aren’t frequent, they may mimic common inflammatory or neoplastic pathologies and could be a diagnostic challenge for the clinician. Case presentation: A 65-year-old woman with a history of Relapsed Refractory Multiple Myeloma (RRMM) presented at our outpatient Otolaryngology department with a four-month history of right-sided aural fullness and a minor episode of watery otorrhea. Otoscopy revealed an abnormal tympanic membrane lesion, and audiological evaluation showed moderate conductive hearing loss. The rest of the examination was normal. A Computed Tomography (CT) scan showed a soft-tissue mass occupying the tympanic membrane with associated temporal bone erosion. The history of RRMM and otoscopic and CT findings were indicative of EMI. After multidisciplinary consultation with a hematologist and radiologist, systemic therapy for relapsed disease was initiated, with consideration of adjunctive radiotherapy. However, the clinical course of the patient deteriorated and she eventually died due to her multiple comorbidities. Conclusions: EMI of the tympanic membrane is a rare entity, among the few reported cases with otologic involvement and it remains a diagnostic “challenge” because it resembles many other otologic conditions. Careful analysis of imaging findings in combination with the patient’s medical history is the key to accurate diagnosis. Early recognition is necessary to guide appropriate management, avoid unnecessary surgical intervention, and enable timely initiation of systemic therapy

## 1. Introduction

Extramedullary involvement (EMI) in patients with multiple myeloma (MM) is most frequently presented in relapsed or advanced stages and is associated with aggressive biological behavior and an unfavorable prognosis [1]. EMI occurs when neoplastic plasma cells proliferate outside the bone marrow microenvironment, whereas MM consists of plasma cell proliferation in the bone marrow [2,3,4]. At the time of diagnosis, EMI in patients with MM is identified in approximately 7–18%, with paraskeletal plasmacytomas (PS) predominating, and at relapse in up to 20% [5]. The management of EMI requires a highly customized and intensive therapeutic strategy that includes systemic hematological treatment, topical interventions, and close follow-up [6,7]. Careful monitoring is required to evaluate the therapeutic response and detect treatment-related or disease-associated relapses [7].

To the best of our knowledge, involvement localized to the tympanic membrane has not been previously described in the literature, with prior reports involving the external auditory canal, middle ear cavity, mastoid, or petrous bone [8,9,10,11,12]. Furthermore, this is a case in which the disease was not limited to the tympanic membrane but also extended to the temporal bone. This feature highlights that Ear-Nose-Throat (ENT) specialists should maintain a high level of suspicion and proceed to imaging, not relying on otoscopy alone, when evaluating such cases. Similarly, hematologists should be alert to the possibility of new disease extension even when presenting symptoms are atypical or nonspecific.

The aim of this report is not only to present an uncommon case of extramedullary involvement of the tympanic membrane with temporal bone erosion in a patient with MM, but also to highlight how it could have been misinterpreted and how the patient’s hematologic history guided the correct diagnosis and management. This case shows the importance of careful analysis of the patient’s medical history in a multidisciplinary setting, which may help avoid unnecessary surgical intervention and support timely initiation of systemic therapy, especiallyin frail and high-risk patients with multiple comorbidities.

## 2. Case Presentation

A 65-year-old woman presented to the outpatient ENT clinic with a four-month history of a sensation of fullness in the right ear. She also mentioned a brief episode of watery otorrhea lasting one day that occurred some days prior to her presentation. She did not have any other otologic symptoms such as otalgia, vertigo, or tinnitus. She had a 5-year known medical history of low-risk IgG-κ MM according to the International Staging System (ISS): Stage I and the Revised International Staging System (R-ISS): Stage I. She was heavily pretreated with Proteasome Inhibitor (PI), Immunomodulatory agent (IMiD), CD38 antibody, ASCT, and she had previous extramedullary relapses involving the brain and right pelvis. She also had a history of arterial hypertension, dyslipidemia, type 2 diabetes mellitus, and hypothyroidism. Her latest laboratory findings until then during this treatment were IgG: 1260 mg/dL, serum κ light chains: 31.1 mg/dL and κ/λ ratio: 25.7. At presentation to our outpatient department, she was receiving third-line treatment with carfilzomib (PI) and selinexor. At that time, laboratory findings showed an IgG level of 1890 mg/dL, a serum free κ light chain level of 99.9 mg/L and κ/λ ratio of 81.1.

During physical examination, no remarkable findings were observed. Vestibular testing was also normal, and neurological examination showed no evidence of facial nerve dysfunction or involvement of other cranial nerves. Otoscopic examination revealed an abnormal mass at the tympanic membrane, without any signs of acute infection. (Figure 1) Audiological testing with a tuning fork and audiogram demonstrated unilateral, moderate conductive hearing loss, with an air-bone gap of approximately 40 dB. Initial differential diagnosis for the tympanic membrane lesion included chronic otitis media, cholesteatoma, granulomatous disease and tympanic membrane carcinoma. However, EMI became the most probable diagnosis once HRCT showed temporal bone erosion, given the patient’s known MM and prior relapses.

High-resolution computed tomography (HRCT) of the temporal bones showed a soft-tissue mass occupying Prussak’s space, accompanied by erosion of the temporal bone (Figure 2). When the patient presented to our outpatient ENT department, her clinical condition had already been significantly compromised by her already existing disease and associated comorbidities. Her overall performance status did not allow for an invasive diagnostic procedure such as biopsy. Based on the patient’s history of RRMM and the clinical and radiological findings, the case was discussed at a multidisciplinary team meeting, including specialists in otolaryngology, hematology, and radiology, where initiation of systemic therapy was preferred over tissue sampling to avoid any delay in treatment. Those findings, in the context of the patient’s known MM, were diagnostic of EMI. Systemic therapy for RRMM was therefore initiated with a BCMA bispecific antibody, with consideration of adjunctive local radiotherapy for symptom control. The patient had her follow-up in the hematology outpatient clinic and our otolaryngology department. Two months later, the patient presented to the hematology emergency department with generalized weakness. She was admitted to the hospital for further management. At that time, immunoglobulin electrophoresis and immunofixation tests showed an IgG immunoglobulin level of 2080, a serum κ light chain level was 125, and a κ/λ ratio of 474. A contrast-enhanced brain CT of 2 mm and 5 mm slice thickness revealed progression of the temporal lesion (Figure 3). Her condition later worsened significantly, and owing to her multiple comorbidities, she died.

## 3. Discussion

Recent expert consensus has supported classifying EMI into two biologically and clinically similar entities: the true Extramedullary Disease (EMD) and the PS (paraskeletal) disease. The first consists of soft-tissue plasmacytomas resulting from hematogenous spread without bone continuity, whereas the second, which represents the predominant pattern, consists of soft-tissue masses arising from skeletal lesions following cortical bone disruption. Our patient has combined features of both entities, a soft-tissue mass involving the tympanic membrane and a temporal bone erosion. While EMI usually affects bones or visceral sites, otologic involvement is considerably rarer. It represents a diagnostic challenge, as lesions of the temporal bone, middle ear, or external auditory canal frequently present with nonspecific clinical and radiological features [8,9]. Previous reports and small case series have characterized temporal bone plasmacytomas as a “great imitator”, given their competence to mimic more common otologic entities such as chronic otitis media, cholesteatoma, inflammatory mastoid disease, or external auditory canal carcinoma, usually causing delayed or incorrect initial diagnosis [8,9,10]. Notably, in the case reported by Aishwarya et al., the intraoperative frozen section was initially diagnosed as a glomus tumor, indicating the diagnostic challenge, with EMI established only after paraffin-embedded histopathological examination. [10] The localization of the lesion could result in a false diagnosis, because temporal bone involvement has been documented predominantly in isolated case reports and limited series [8,9,10,12]. In this context, careful analysis of imaging findings together with the patient’s clinical history and the multidisciplinary setting is of great importance, particularly when biopsy confirmation is delayed or not feasible [11].

In MM patients, an apparently inflammatory tympanic membrane lesion that fails to fit the usual clinical pattern should prompt cross-sectional imaging and consideration of EMI. In our case, the central lesion of the tympanic membrane was not at first indicative that it would be associated with the temporal bone. The patient’s known history of multiple myeloma was carefully correlated with the clinical suspicion of disease relapse, leading us to select CT as the imaging method, which revealed the bone erosion. At initial presentation, the otoscopic appearance was suggestive of an inflammatory process. However, the HRCT findings favored EMI over an inflammatory etiology: the extent of bone destruction was extensive, with punched-out lesions, and involved the cortical bone rather than being confined to the mastoid air cells. Furthermore, the pattern of soft-tissue involvement accompanying the bone erosion was not typical of cholesteatoma or other inflammatory disease. All these factors enabled prompt diagnostic evaluation and guided the selection of an appropriate therapeutic strategy, illustrating the importance of individualized, patient-specific treatment planning in the management of EMI. Recognition of this association is essential, as accurate diagnosis directly influences subsequent therapeutic strategy and prognostic evaluation [1].

Management of extramedullary plasmacytomas involving the temporal bone and tympanic membrane is a challenging topic that requires a multidisciplinary approach that equilibrates systemic disease control with the preservation of audiological and vestibular function. Our patient’s case was discussed in a multidisciplinary setting and initiation of systemic therapy rather than pursuing local procedures was chosen because the lesion reflected active, progressive systemic disease that had already failed multiple prior lines of therapy, taking into consideration the anatomical complexity of the temporal bone and the patient’s clinical status. Adjunctive radiotherapy was considered owing to the marked radiosensitivity of plasma cell neoplasms and its potential for local tumor control and symptom relief, but was not pursued due to rapid clinical deterioration [11,13]. Surgical intervention was not selected, as it is generally reserved for histopathological confirmation or selected palliative indications, such as decompression of cranial nerves or the middle ear space. Given the patient’s poor general condition and the need to preserve essential temporal bone anatomy and function, neither indication applied in this case [4,7,13].The principal contribution of this report is not only the rarity of the anatomical location of RRMM, but also the diagnostic pathway and the cooperation of multiple specialists. Both ENT and hematologist specialists should be aware that in patients with known RRMM, benign otologic symptoms may represent disease relapse rather than primary otologic pathology. In our patient, correlation of the clinical history with HRCT findings and multidisciplinary discussion allowed timely recognition of EMI despite the absence of histopathological confirmation.

A limitation of this report is the absence of histopathological confirmation, which remains the gold standard approach for temporal bone and tympanic membrane masses. This case represents an exception to standard practice, justified by the combination of strong clinical and radiologic evidence together with the patient’s history of active extramedullary RRMM. When the patient presented to our outpatient ENT clinic, her overall clinical condition, which had already been compromised due to her underlying disease, did not allow for an invasive diagnostic procedure. Given the patient’s history of active RRMM, prior extramedullary relapses, the otoscopic and CT findings, and the patient’s frailty, the case was discussed in a multidisciplinary setting where it was judged appropriate to avoid an invasive procedure such as biopsy and to initiate systemic therapy. This approach should not be interpreted as a general recommendation to omit histopathological confirmation in similar lesions in otherwise fit patients, for whom biopsy remains the standard diagnostic step.

Systemic therapy continues to be the gold standard of treatment and needs to be aggressive, despite the generally poor prognosis. Given disease progression while on carfilzomib and selinexor, and an extensive prior treatment history including PI-, IMiD-, and CD38 antibody-based regimens, ASCT, and prior extramedullary relapses, a BCMA-directed bispecific antibody was selected as the next-line option at the time of EMI diagnosis, consistent with current approval criteria for heavily pretreated RRMM patients with prior exposure to these drug classes [14]. However, reported responses are typically short-lived, with a median duration of less than 4 months [2,15,16].

The prognosis of extramedullary involvement is unfavorable, with survival typically less than 6 months in relapsed cases [15,17]. Patients with paraskeletal disease have better survival outcomes than those with true EMD arising from hematogenous dissemination [2,18]. This poor prognosis was reflected in our patient’s already fragile clinical condition, which was further burdened by the new relapse involving the tympanic membrane and temporal bone. Her subsequent death within two months likely reflected the combined effect of this relapse and her multiple underlying comorbidities, rather than the EMI alone. Due to this unfavorable prognosis, close follow-up by a multidisciplinary team is essential for the early detection of relapse or treatment resistance.

## 4. Conclusions

EMI of RRMM in the tympanic membrane and mastoid bone is rare and demonstrates how challenging its diagnosis could be. Otologic symptoms and findings may mimic inflammatory or neoplastic conditions of the ear, thereby delaying the diagnosis. This case highlights that, in patients with known MM—especially RRMM with prior extramedullary disease—atypical tympanic membrane or middle ear lesions should raise suspicion for EMI and prompt appropriate imaging and multidisciplinary evaluation. Early recognition of such lesions may avoid unnecessary surgery and allow timely initiation of systemic treatment, even when histologic confirmation is not feasible because of clinical frailty.

## Figures and Tables

**Figure 1:**
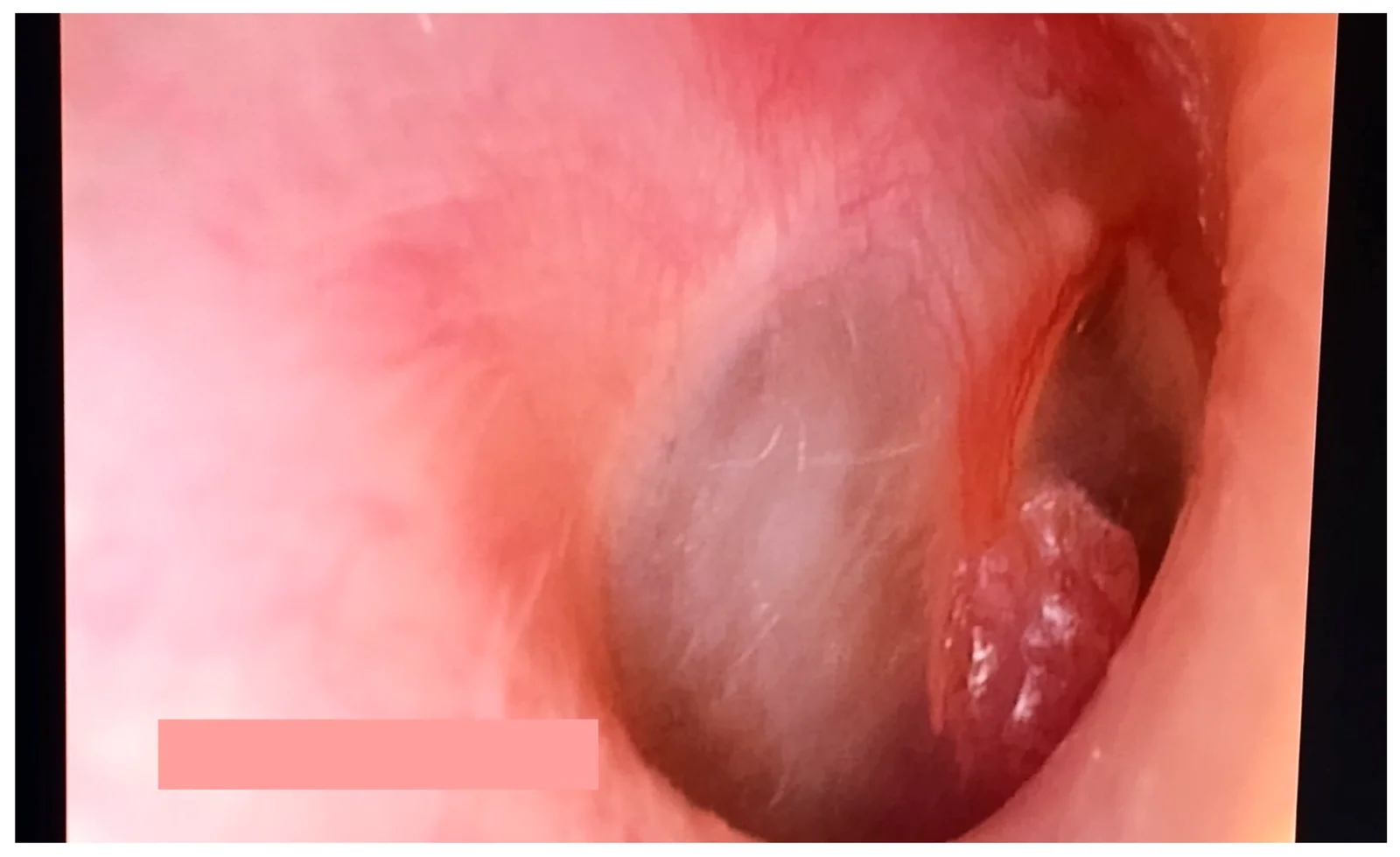
Irregular mass arising from the tympanic membrane without signs of acute infection.

**Figure 2:**
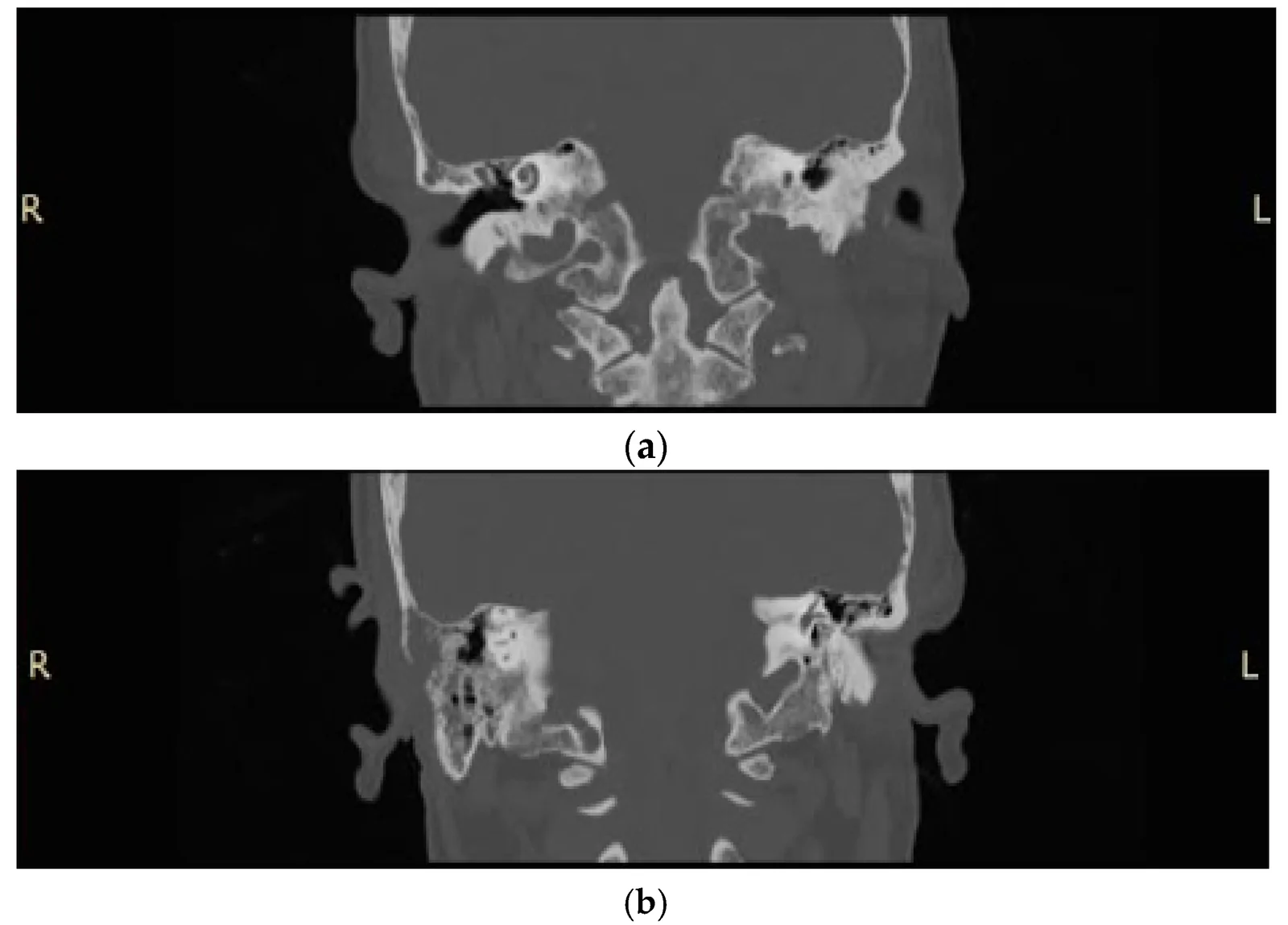
(**a**,**b**): HRCT of the temporal bones, obtained at initial presentation, showing a soft-tissue mass of Prussak’s space (**a**) with adjacent bone erosion (**b**).

**Figure 3:**
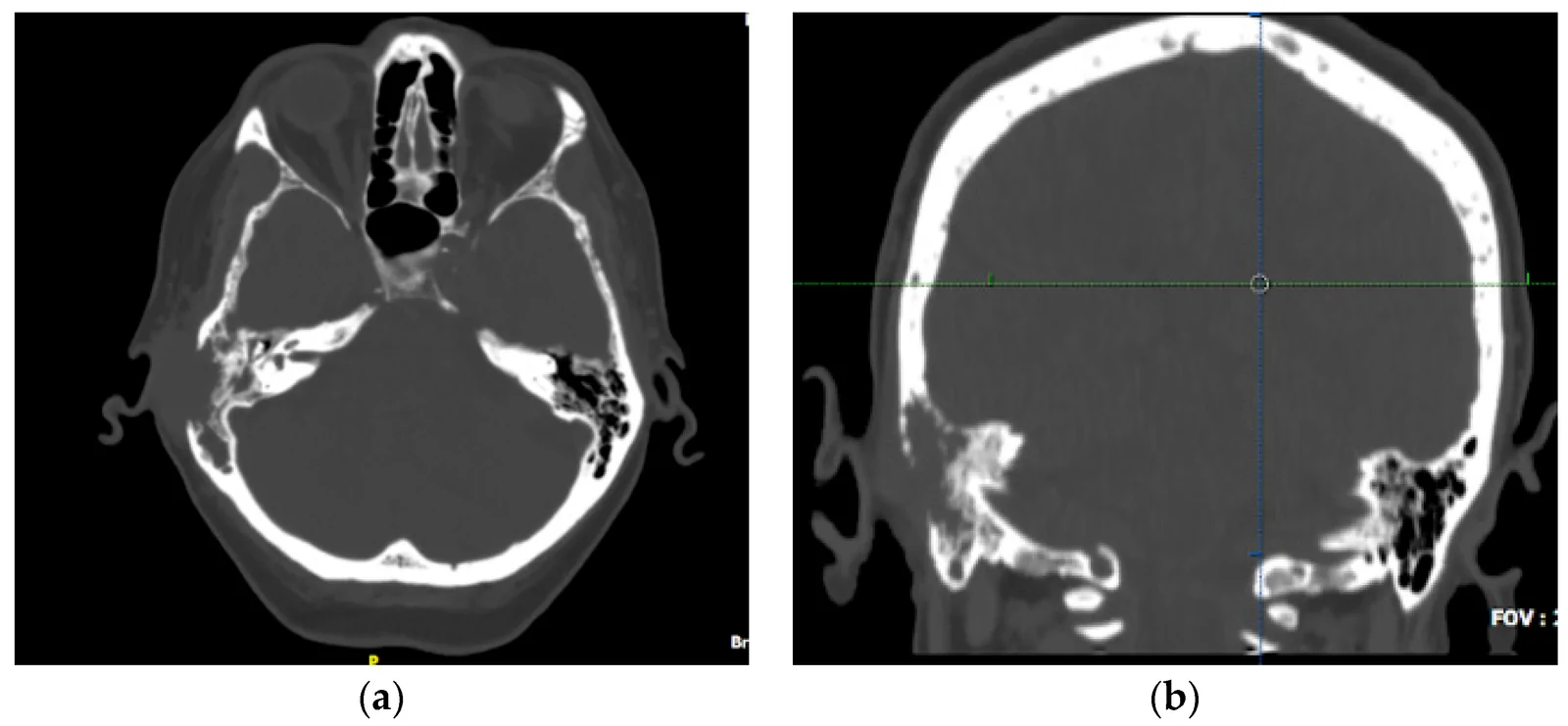
(**a**,**b**): Follow-up CT (**a**,**b**) obtained two months later, showing progression of disease in the right temporal bone, compared to Figure 2.

## Data Availability

The original Contributions presented in the study are included in the article. Further inquiries can be directed to the corresponding author.

## Abbreviations

The following abbreviations are used in this manuscript:

EMI	Extramedullary Involvement
MM	Multiple Myeloma
RRMM	Relapsed Refractory Multiple Myeloma
CT	Computed Tomography
HRCT	High Resolution Computed Tomography
EMD	Extramedullary Disease
PS	Paraskeletal plasmacytomas
ASCT	Autologous Stem Cell Transplantation
ENT	Ear-Nose-Throat
ISS	International Staging System
R-ISS	Revised International Staging System
PI	Proteasome Inhibitor
IMiD	Immunomodulatory Agent
